# Lifestyles, environmental and phenotypic factors associated with lip cancer: a case–control study in southern Spain

**DOI:** 10.1038/sj.bjc.6600975

**Published:** 2003-05-27

**Authors:** E Perea-Milla López, R M Miñarro-del Moral, C Martínez-García, R Zanetti, S Rosso, S Serrano, J F Aneiros, A Jimenez-Puente, M Redondo

**Affiliations:** 1 Unidad de investigación, Hospital Costa del Sol, Ctra Nacional 340, km 187, Marbella, 29600 Málaga, Spain; 2 Escuela Andaluza de Salud Pública, Campus Universitario de Cartuja, 18080 Granada, Spain; 3 Registro dei Tumouri per il Piemonte e la Valle d'Aosta, via S. Francesco da Paola 31, 10123 Turin, Italy; 4 Servicio de Dermatología, Hospital Clínico San Cecilio, Avda Dr. Olóriz, 18012 Granada, Spain; 5 Servicio de Anatomía Patológica, Hospital Clínico San Cecilio, Avda Dr Olóriz, 18012 Granada, Spain

**Keywords:** lip cancer, risk factors, case–control studies, sun exposure, tobacco, alcohol, warts

## Abstract

The aim of this study was to identify factors related to lip cancer (LC) considering individual characteristics and sociodemographic factors. A case–control study was carried out in the province of Granada (Andalusia, southern Spain). The cases were 105 males with squamous-cell carcinoma of the lip, diagnosed between 1987 and 1989 (aged 20–70 years) and identified by means of a population-based Cancer Registry. As controls, a randomised populational sample of 239 males, stratified by age, was used. Multiple logistic regression analysis showed that risk factors are lifetime cumulative tobacco consumption and alcohol consumption. An interaction was found between alcohol consumption and the smoking habit (leaving the cigarette on the lip): OR=23.6; 95% CI: 3.9–142.0. Other risk factors identified are clear eyes (OR=3.5; CI: 95% 1.5–8.0), sun exposure early in life and cumulative sun exposure during outdoor work (OR=11.9; 95%: CI: 1.3–108.9), and skin reaction to sun exposure (Fitzpatrick levels). Another interaction was found between skin reaction and a previous history of common sporadic warts (OR=4.4; 95% CI: 1.01–19.1). We conclude that LC is related to phenotype, skin reaction to sun exposure, cumulative and early sunlight exposure, and tobacco and alcohol consumption, as well as a low educational level. Leaving the cigarette on the lip is predictive of LC risk irrespective of cumulative tobacco consumption.

According to information from the population-based Cancer Registry in Granada (Andalusia, southern Spain), lip cancer (LC; ICD9-MC# 140) was the seventh most frequent site in incidence in males during the period 1988–1992, one of the highest such rates in the world (age standardised rate: 12.0 × 100 000 males per year) together with the Canadian and Australian registries. In Spain, the population-based registries of Murcia and Zaragoza also reported high figures, although lower than Granada (Parkin *et al*, 1997).

Lip cancer is more frequent in males than in females, and in white populations (Muir and Weiland, 1995; Parkin *et al*, 1997). During recent decades, a decline in incidence rates has been reported in most registries in the world (Boyle *et al*, 1995). Numerous risk factors have been related to LC, although evidence for many of them remains controversial (de Visser and Van der Waal, 1998). It seems reasonable that LC, because of its anatomical location, may share some risk factors with skin tumours (like sun exposure and phenotype) and oral cavity and pharynx neoplasms (like alcohol intake and tobacco consumption). Lip cancer has been related to sun exposure (International Agency for Research on Cancer, 1986) in different descriptive studies of migrants (McCredie and Coates, 1989; Steinitz *et al*, 1989) and in several case-control studies, measured with proxies like outdoor activities/work (Keller, 1970; Spitzer *et al*, 1975; Lindqvist, 1979; Dardanoni *et al*, 1984), although potential confounding by tobacco and alcohol (both potentially associated with LC) cannot be discounted (International Agency for Research on Cancer, 1992). Other factors that have been related to LC are low socioeconomic status (Williams and Horm, 1977; Lindqvist *et al*, 1981), viral infections, family predisposition and immunosuppression (de Visser and van der Waal, 1998). The aim of this study was to determine the association between male LC and tobacco consumption and alcohol intake, as well as different environmental and socioeconomic factors, in a high-risk area for LC represented by Granada (Spain).

## METHODS

A population-based case–control study was carried out in the province of Granada (Spain), including all incident cases of LC in males aged 20–70 years, diagnosed between 1987 and 1989, with histological confirmation and resident in the province. Cases were identified through the population-based Granada Cancer Registry.

Controls were drawn from the male population of the province, and consisted of a random sample obtained through the Local Population Registry of 1986. Two controls for each case were considered, taking into account the distribution of cases by age (5-year stratified frequency matching).

Definition and ascertainment of exposure: cases and controls were interviewed by previously trained interviewers using the standardised questionnaire of the European Helios study on nonmelanoma skin cancer (Zanetti *et al*, 1996). This questionnaire was modified in order to complement the information on tobacco consumption and to include alcohol consumption in detail. A complete smoking history was obtained, including the average number of cigarettes smoked daily, age at start and duration of smoking, type of tobacco (blond, black), use of filter cigarettes, leaving the cigarette on the lip while smoking, taking into account possible variations in smoking habits. The lifetime cumulative number of cigarettes smoked was computed and analysed according to the quartiles of the controls. The first category of smokers considered comprised those who had smoked less than 135 560 cigarettes during their life, the equivalent of about 1 pack per day for 18.5 years.

The information collected on alcohol intake was similar to that for tobacco consumption (age at start, duration, type of drink and daily amount). The lifetime cumulative index for alcohol intake was derived from these data, after transforming each type of drink into the corresponding ethanol content.

The Helios questionnaire covered phenotype characteristics such as hair colour, eye colour, Fitzpatrick levels (in a range of I–IV, with the Fitzpatrick phototype level I being ‘never tan, always burn’ and level IV being ‘always tan, never burn’) (Fitzpatrick *et al*, 1974), sociodemographic characteristics (i.e. educational level, occupation), lifelong sun exposure (in diverse activities during work, leisure and holiday periods, and specifying the season of the year) and dermatological history. An index of lifetime cumulative sun exposure was computed as previously described (Rosso *et al*, 1996), and for various activities. Two seasons were defined: a summer period, from April to September, and a winter season. The two indexes, both individually and combined (total cumulative sun exposures), were considered for the analyses, although when there was no distinct difference between leisure and holiday sun exposure, only the results for total cumulative variables are shown.

Statistical analysis was performed through logistic regression. Continuous variables (cumulative indexes for tobacco, alcohol consumption and sun exposure) were categorised taking into account the quartiles of the exposed controls (Clayton and Hills, 1993). When the reference category (nonexposed) had few subjects, an analysis was performed after changing the reference category and, if the results were consistent, the nonexposed group remained as the reference. In the case of categories with similar coefficients and standard errors, these categories were combined. A multiple logistic regression model was built, which included new variables relative to statistical criteria (*P*<0.15) and confounding criteria (changes in odds ratio [OR] >10%). The diagnosis of the model considered the Cook extreme distances.

## RESULTS

*Recruitment*: A total of 129 potential cases were considered. Three cases (2.3%) refused to be interviewed, and interviews could not be conducted in 21 cases (four dead, 15 untraceable and two disabled). Finally, 105 cases were included in the study (response rate 81.4%). Of the 340 potential individuals in the control group, 24 subjects refused to be interviewed (7.1%) and 77 interviews could not be conducted (16.8% untraceable, mainly due to errors in the Population Registry). In all, 239 control subjects took part in the study (response rate 70.3%). Table 1

**Table 1:** Distribution of cases and controls by age group

**Age group (years)**	**Cases *n* (%)**	**Controls *n* (%)**
20–40	6 (5.7)	12 (5.0)
41–45	4 (3.8)	8 (3.4)
46–50	10 (9.5)	16 (6.7)
51–55	18 (17.1)	32 (13.4)
56–60	17 (16.2)	42 (17.6)
61–65	26 (24.8)	55 (23.0)
66–70	24 (22.9)	74 (30.9)
		
Total	105	239

shows the distribution of cases and controls by age group.

All LC cases were of squamous-cell carcinoma (SCC) on the lower lip in men. Regarding tobacco consumption, only two LC cases had never smoked, while nonsmokers accounted for 16.3% of the controls. The case group had started to smoke earlier than the controls: 61.2% of the smoker cases began at 15 years or less, *vs* 47.8% of the controls. Exsmokers accounted for 22.9% of the case group and 36.4% of the controls. The median age of giving up smoking was similar in both groups of exsmokers (52 and 50 years old for cases and controls, respectively), although the controls started to give up younger (range 31–68 years in cases, 15–68 years in controls). Only four cases and eight controls said they smoked cigars and, of these, all but three controls combined cigars and cigarette smoking. Pipe smoking was only reported by one participant in each group, both of whom also smoked cigars and cigarettes.

Considering the type of tobacco (Table 2

**Table 2:** Distribution by variables not included in the logistic regression model (OR adjusted by age)

**Variables**	**Cases *n* (%)**	**Controls *n* (%)**	**OR (95% CI)**
Type of tobacco			
Nonsmoker	2 (1.9)	39 (16.3)	1 (reference)
Only blond	6 (5.7)	35 (14.6)	2.3 (0.5–10.0)
Only black	91 (86.7)	143 (59.8)	11.2 (3.3–37.5)
Blond and black	5 (4.8)	15 (6.3)	5.3 (1.1–24.7)
			
*Filter use*			
Nonsmoker	2 (1.9)	39 (16.3)	1 (reference)
With filter	39 (37.1)	93 (38.9)	8.1 (1.9–34.8)
Without	9 (8.6)	32 (13.4)	6.2 (1.2–30.7)
Both types	55 (52.4)	75 (31.4)	15.1 (3.5–65.2)
			
*Hair colour*			
Dark	85 (81.0)	205 (85.8)	1 (reference)
Fair	20 (19.0)	34 (14.2)	1.4 (0.8–2.6)
			
*Leisure time sun exposure*			
Non exposed	35 (33.3)	74 (31.0)	1 (reference)
First quartile (<1.2 weeks)	23 (21.9)	41 (17.2)	1.2 (0.6–2.3)
Second quartile (<4.5 weeks)	24 (22.9)	41 (17.2)	1.2 (0.6–2.3)
Third quartile (<14.3 weeks)	17 (16.2)	42 (17.2)	0.8 (0.4–1.7)
Fourth quartile (⩾14.3 weeks)	6 (5.7)	41 (17.2)	0.3 (0.1–0.9)
			
*Holiday sun exposure*			
Non exposed	81 (77.1)	148 (61.9)	1 (reference)
First quartile (<0.5 weeks)	15 (14.3)	23 (9.6)	1.2 (0.6–2.3)
Second quartile (<2.9 weeks)	4 (3.8)	22 (9.2)	0.3 (0.1–0.97)
Third quartile (<11.2 weeks)	4 (3.8)	23 (9.6)	0.3 (0.1–0.97)
Fourth quartile (⩾11.2 weeks)	1 (1.0)	23 (9.6)	0.1 (0.01–0.6)
			
*Farming, fishing, forestry*			
Never	15 (14.3)	79 (33.0)	1 (reference)
At some time	90 (85.7)	160 (67.0)	3.1 (1.7–5.7)
			
*Services*			
Never	90 (85.7)	153 (64.0)	1 (reference)
At some time	15 (14.3)	86 (36.0)	0.3 (0.2–0.6)
			
*Lip herpes*			
No	40 (38.1)	127 (53.1)	1 (reference)
Sporadic	38 (36.2)	71 (29.7)	1.7 (1.0–2.9)
Chronic	27 (25.7)	41 (17.2)	2.2 (1.1–3.8)
			
*Herpes zoster*			
No	90 (85.7)	198 (82.9)	1 (reference)
Sporadic	14 (13.3)	36 (15.1)	0.9 (0.4–1.7)
Chronic	1 (1.0)	5 (2.1)	0.4 (0.1–3.8)

), the most common pattern was the exclusive consumption of black tobacco in both groups, although in greater proportions among cases than among the controls (crude OR against nonsmokers=11.2, 95% CI: 3.3–37.5). The OR for smokers with or without filter was similar in a crude analysis, while the corresponding estimate for smokers of both types of cigarettes was twice as high as that of the former group. When we adjusted the values by the lifetime cumulative number of cigarettes smoked, this association disappeared.

Fair hair was not statistically associated with the risk of LC (OR=1.4; 95% CI: 0.8–2.6). In terms of occupational exposures, about 90% of cases had, at some time in their life, been engaged in fishing, agricultural or forestry work, whereas only 67% of the controls had been engaged likewise. Similar percentages were found for industrial workers. In the control group, the services sector was more frequent than in cases.

Lifetime exposure to sun during leisure and holidays revealed a protective effect in the highest quartiles. This association disappeared when the figures were adjusted by number of years of study.

Lip herpes antecedents were more frequent among the case group, although the association disappeared after adjustment by other variables. No association was found with herpes zoster.

The multivariate logistic regression model for LC is shown in Table 3

**Table 3:** Multivariate logistic model, excluding interactions (adjusted by cumulative alcohol intake, leaving the cigarette on the lip, warts and phototype, and age)

	**Cases *n* (%)**	**Controls *n* (%)**	**Crude OR (95% CI) (adjusted by age)**	**Adjusted OR (95% CI)**
*Tobacco*				
Nonsmoker	2 (1.9)	39 (16.3)	1 (reference)	1 (reference)
First quartile (<135 560 cigarettes)	12 (11.4)	48 (20.1)	5.0 (0.98–47)	2.4 (0.4–13.1)
Second quartile <274 540 cigarettes)	25 (23.8)	51 (21.3)	9.6 (2.1–86.9)	7.7 (1.4–40.7)
Third quartile (<455 402 cigarettes)	38 (36.2)	51 (21.3)	14.5 (3.3–129.7)	9.25 (1.8–48.3)
Fourth quartile (⩾455 402 cigarettes)	28 (26.7)	50 (20.9)	10.9 (2.5–98.7)	5.32 (0.94–30.1)
				
*Eye colour*				
Black-dark brown	16 (15.2)	95 (39.8)	1 (reference)	1 (reference)
Hazel, green, blue, grey	89 (84.8)	144 (60.3)	3.7 (2.0–7.1)	3.5 (1.5–8.0)
				
*Sun exposure, outdoor work (April–September)*				
Nonexposed	1 (1.0)	37 (15.5)	1 (reference)	1 (reference)
First quartile (<35.4 weeks)	21 (20.0)	50 (20.9)	16.1 (2.3–114.8)	12.6 (1.2–132.4)
Second quartile (<68.6 weeks)	29 (79.1)	51 (21.3)	22.9 (3.2–162.9)	11.7 (1.2–115.5)
Third quartile (<122.6 weeks)	23 (21.9)	51 (21.3)	18.7 (2.6–133.7)	12.7 (1.4–118.0)
Fourth quartile (⩾122.6 weeks)	31 (29.6)	50 (20.9)	25.2 (3.6–179.1)	11.9 (1.3–108.9)
				
*Age at first sunburn*				
No burn	99 (94.3)	203 (84.9)	1 (reference)	1 (reference)
⩽15 years old	1 (1.0)	3 (1.3)	0.7 (0.07–6.6)	14.6 (0.8–255.1)
>15 years old	5 (4.8)	33 (13.8)	0.3 (0.1–0.8)	0.1 (0.03–0.6)
		/		
*Years of study*				
>3	29 (27.6)	138 (57.7)	1 (reference)	1 (reference)
⩽3	76 (72.4)	101 (42.3)	3.4 (2.1–6.1)	2.2 (1.1 – 4.4)

, 4

**Table 4:** Multivariate logistic regression model (continuation)

	**No warts**	**Sporadic warts**	**Chronic warts**
Phototype IV	1 (reference)	0.9 (0.2–3.7)	6.2 (0.6–67.5)
	8 cases/23 controls	9 cases /16 controls	3 cases/3 controls
Phototype III	0.6 (0.2–2.3)	0.9 (0.2–4.6)	Undefined
	9 cases/29 controls	5 cases/8 controls	0 cases/7controls
Phototype II	0.4 (0.1–1.1)	1.12 (0.3–4.2)	1.04 (0.3–4.3)
	13 cases/75 controls	11 cases/24 controls	10 cases/12 controls
Phototype I	2.8 (0.8–9.8)	4.4 (1.01–19.1)	9.82 (0.5–177.0)
	18 cases/31 controls	14 cases/8 controls	5 cases/2 controls

OR for interaction terms 'Warts and phototype'; 95% CI, number of cases and controls in each cell (one control with missing value for phototype with chronic wart); adjusted by age, tobacco, alcohol, leaving the cigarette on the lip, study level, eye colour and sun exposure during outdoor work in summer

and 5

**Table 5:** Multivariate logistic regression model (continuation)

	**Nondrinkers**	**Alcohol (first quartile)**	**Alcohol (second quartile)**	**Alcohol (third quartile)**	**Alcohol (fourth quartile)**
		**(<217.281 cm^3^) ethanol**	**<415 606 cm^3^ ethanol**	**<853 114 cm^3^ ethanol**	**⩾853 114 cm^3^ ethanol**
Do not leave the cigarette on the lip	1 (reference)	2.0	4.0	4.6	2.2
	3 cases/29 controls	(0.40–12.0)	(1.01–23.0)	(1.2–26.1)	(0.4–11.2)
		8 cases/41 controls	17 cases/41 controls	19 cases/40 controls	8 cases/40 controls
Leave the cigarette on the lip	Undefined	9.7	9.7	13.6	23.6
	0 cases/10 controls	(1.8–64.0)	(1.8–64)	(2.8–84.0)	(3.9–142.0)
		9 cases/9 controls	9 cases/9 controls	14 cases/10 controls	18 cases/10 controls

OR for interaction terms: cumulative alcohol intake and leaving the cigarette on the lip; number of cases and controls in each cell and 95% CI; adjusted by age, tobacco, eye colour, warts, sun exposure during outdoor work in summer, age at first sunburn and years of study

; it included as predictors the variables related to phenotype (eye colour), skin reaction to sun exposure (phototype measured as Fitzpatrick levels), previous common warts, cumulative and early sunlight exposure (cumulative exposure to the sun in summer work and age at first sunburn), cumulative tobacco and alcohol consumption (amounts of alcohol and tobacco consumed in a lifetime, and the habit of leaving the cigarette on the lip while smoking), and a proxy of educational levels.

Cumulative exposure to the sun in summer work (Table 3) presents increases from moderate exposures (first quartile). Sun exposure during outdoor work was present in 99.1% of the case group but only in 84.5% of the controls (the first quartile would be equivalent to working 8 h/day for 5 days/week during a 24-week period (April–September) for 6 years, approximately).

The age at first sunburn was also related to the risk for LC; people who had experienced their first sunburn after age 15 years, as compared to those who had never experienced a sunburn, presented a lower risk.

Subjects with clear eyes (hazel to grey), and those with fair hair, were more common among the case group than among the controls. The distribution of skin reaction to sun exposure showed that more sensitive individuals (Fitzpatrick level I: never tan, always burn) were more common among the case group.

A higher Fitzpatrick level implies increased risk when the skin is more sensitive, and its effect depends on the presence or absence of common warts (Table 4). In fact, the increased risk for LC was only found in very sensitive skin (Fitzpatrick level I) and was two-fold in the presence of sporadic warts, compared with no warts (*P*<0.05). No significant differences were detected in those with antecedents of chronic warts. In fact, common antecedents of warts were more frequent among the case group: sporadic common warts were found in 37.1% of the case group, but only in 23.4% of the controls; similarly, chronic warts were also more frequent in the case group, although in a smaller proportion.

The amounts of alcohol and tobacco consumed in a lifetime are also significantly associated. However, not only the amount of tobacco consumed but also the pattern of smoking is related to this type of cancer. The habit of leaving the cigarette on the lip while smoking increases the risk. In addition, these risks are not independent: we found an interaction (*P*<0.05) between the amount of alcohol consumed and the habit of leaving the cigarette on the lip (Table 5). The OR associated with an excess of accumulated alcohol (fourth quartile) was 23.6 (95% CI: 3.9–142.0) in those who frequently leave the cigarette on their lip.

Leaving the cigarette on the lip while smoking was a relatively frequent practice among the case group (48.5% of the latter *vs* 23% of the controls). After allowance for cumulative tobacco consumption, neither the type of tobacco nor the use of a filter could be included in the logistic model. Leaving the cigarette on the lip was predictive of LC risk irrespective of cumulative tobacco consumption. Furthermore, the effect of leaving the cigarette on the lip varied with the level of alcohol consumption, but no interaction was found between cumulative tobacco consumption and alcohol intake. Alcohol consumption was more frequently reported by the case group than by the controls (98 *vs* 89%), with greater differences for higher cumulative doses. Alcohol intake started early in life in both groups (aged 18 years or less: 66% of the case group and 60% of the controls).

Having studied less than 3 years was associated with an almost two-fold risk of developing LC (72.4% of the case group *vs* 42.3% of the controls).

The six observations with Cook distances of 0.5 and above only presented a loss of significance in the ‘first sunburn before age 15 years’ category.

## DISCUSSION

In our study, there is evidence to confirm the independent effect of exposure factors related to the oral cavity (alcohol, tobacco), and of those related to skin cancer, like cumulative sunlight and susceptibility (phototype, eye colour). The educational level, measured by means of years of study and sunlight exposure during leisure time, is also an important predictor.

The nonresponse rate was reasonably low for a population-based study. Most of the untraceable controls were due to wrong or inexistent addresses in the Population Registry. Outpatient treatment, especially in private clinics, could represent a source of underdetection of cases, with a socioeconomic bias. However, the Cancer Registry of Granada has access to the clinical registries of private dermatologists and anatomopathologists.

Other methodological issues that complicate the study of LC include the misclassification of lip-skin neoplasms in this site instead of as skin, or *vice versa*, which could distort comparability values. Nevertheless, LC is defined as a cancer that affects the red border and adjacent mucosa of the lip, excluding cancers on the skin of the lip. It usually appears on the lower lip with a histological pattern of SCC, while skin cancer is mainly basal-cell carcinoma (BCC). But although most skin cancers may be BCCs, the number of SCC nonmelanoma skin cancers may be considerable and greater than the number of LC per year. To minimise this bias, only cases with histopathological confirmation were included in the study. On the other hand, the usual sources of information on cancer (mortality and incidence data) are difficult to interpret for LC as long as this is normally considered within the group of tumours of the oral cavity and pharynx, which encompasses very heterogeneous neoplasms (McMichael, 1983), although some risk factors are common to many of these.

Habits of great prevalence, such as alcohol and tobacco consumption, are associated with the risk of LC. Also noticeable is the finding of the interaction between alcohol consumption and the habit of leaving the cigarette on the lip while smoking, which is independent of the cumulative amount of tobacco smoked. The effect of alcohol (at moderate/intermediate or excessive levels of consumption) is more important in subjects with this habit. This is coherent with the interaction between alcohol and tobacco found in cancer of the oral cavity and pharynx (Franco *et al*, 1989; Garrote *et al*, 2001).

The above findings suggest that carcinoma mechanisms on the lip may, to some extent, be different from those produced in the generation of other cancers of the oral cavity, pharynx and larynx (Brennan *et al*, 1995). The association between alcohol, tobacco and the habit of leaving cigarettes on the lip while smoking is consistent with previous findings that related tobacco with LC and with oral cancer (Lindqvist, 1979). From a preventive point of view, the message to smokers is that they should stop smoking and moderate alcohol consumption.

Exposure to ultraviolet radiation was measured by lifelong cumulative exposure to sunlight, distinguishing exposures at different times and during different activities. Accumulated sun exposure during work is associated with a constant level of exposure over the years, and is a predictor for LC (all quartiles). This is consistent with previous findings concerning the prevalence of agricultural, forest and fishery tasks in relation to this type of cancer. It is also coherent with the fact that LC is rare on the upper lip (de Visser and van der Waal, 1998). The results of the European study Helios (a multicentre case–control study on skin cancer) suggest a differential behaviour of accumulated sun during leisure and work time, depending on the type of skin cancer (leisure exposure for BCC and work exposure for SCC), similar to the findings for melanoma (Armstrong, 1988; Kricker *et al*, 1995; Rosso *et al*, 1996).

On the other hand, and paradoxically, accumulated sun in leisure, sports and holidays on the beach seems to have a protective effect, both in winter and summer. However, this association disappears after adjustments for years of study, which constitutes a direct indicator of socioeconomic levels. This strengthens the reliability of the interpretation of accumulated sun in leisure as a socioeconomic indicator (Pion *et al*, 1995).

Ultraviolet radiation exposure is also associated with a young age at the time of occurrence. There is a protective effect if sunburn occurs after this age, which suggests a possible role of early exposures (Zanetti *et al*, 1992).

The relation between lifetime accumulated sun and LC could be influenced by a certain degree of bias in the classification. Nevertheless, we avoided questions about exposures on specific sites (for instance, on the site of the tumour); the interviewers did not know the hypothesis of the study and were distributed at random among cases and controls.

Genetic susceptibility to sunlight radiation, measured by eye colour and Fitzpatrick level, has an independent and relevant role. The finding of phenotype characteristics as risk factors (clear eyes, little tanning ability and sunburns when exposed to sun) allows us to identify population groups that are at a higher risk of suffering this cancer. In addition, these indicators are easily measurable, which facilitates the design of prevention messages in the future.

The independent role of lifetime accumulative exposure to sunlight at work defines a population at high risk of cancer. This risk can be alleviated by preventive policies. Simple protection measures (covering exposed sites while working, wearing headgear or using other ways to protect the head and face) would lead to a significant reduction in new cases. The risk associated with excessive exposure to sunlight during childhood (that which surpasses the regenerative tissue capacity), measured as early sunburns, points also to the importance of protecting children by avoiding the hours of greatest radiation, and protecting exposed sites. These findings are consistent with previous studies that related outdoor work exposure to a high risk of LC (Keller, 1970; Spitzer *et al*, 1975; Lindqvist, 1979), and are coherent with the well-known distribution of LC in white people living in sunny areas and with the protective effect of lipstick in women (Pogoda and Preston-Martin, 1996). A possible source of underestimation of the effect of sun exposure and phenotype is the fact that adjusting for sun exposure and phenotype can be difficult, if people who easily burn intentionally avoid sun exposure.

The finding of an interaction between phototype and antecedents of sporadic warts suggests a possible role of viral agents, probably human papillomavirus (International Agency for Research on Cancer, 1995; Franceschi *et al*, 1996) and/or immunodeficiency. The possible misclassification by actinic lesions can be ruled out; we should take into account that sporadic warts (the only ones that cannot be misclassified because actinic lesions do not disappear) are associated with the risk of LC. Furthermore, there is evidence of a causal relation in cervix cancer and a high prevalence of viral infection in the tumour in the case of oral cancer, even in nonmelanoma skin cancer (Shamanin *et al*, 1996).

We should also note the association between low educational level and LC, even after adjusting for alcohol, tobacco and phenotype characteristics, and the other risk factors. The educational level can be considered a proxy of socioeconomic status, and is probably associated with occupational or nutritional exposures (the latter not considered in this study). This association is consistent with the low socioeconomic level of the province and the high incidence of LC reported by the Registry of Cancer of Granada, and also with the high prevalence of sensitive phenotypes and lifestyles (tobacco and alcohol consumption). The association between LC and a low socioeconomic level was found in a previous study based on southern Europe (Dardanoni *et al*, 1984).

Further studies are needed to seek the definitive characterisation of the risk factors detected, as well as the identification of other factors that are probably involved. In this sense, the present study suggests a possible hypothesis for future verification: the finding of an association with warts and clinical antecedents in any part of the body suggests the possible implication of variables related to viral components and/or immunosuppression (Dinehart *et al*, 1990; King *et al*, 1995). Finally, the risk associated with high alcohol intake may be related, at least in part, to certain nutritional deficits not explored by this study (Franceschi *et al*, 1991).
